# Prevalence of new and known species of haemoparasites in feral pigeons in northwest Italy

**DOI:** 10.1186/s12936-015-0617-3

**Published:** 2015-03-03

**Authors:** Frine Eleonora Scaglione, Paola Pregel, Francesca Tiziana Cannizzo, Antón Davìd Pérez-Rodríguez, Ezio Ferroglio, Enrico Bollo

**Affiliations:** Dipartimento di Scienze Veterinarie, Università degli Studi di Torino, Via L. da Vinci 44, 10095 Grugliasco, Italy; Departamento de Zoología y Antropología Física, Universidad Complutense de Madrid, José Antonio Nováis 2, E-28040 Madrid, Spain

**Keywords:** Feral pigeons, *Columba livia*, Haemoparasites, *Haemoproteus* spp., *Leucocytozoon* spp, *Plasmodium* spp

## Abstract

**Background:**

Haemoparasites in feral pigeons have been studied in several countries but no data are available from Italy. The aim of this work was to evaluate the prevalence and diversity of *Haemoproteus* spp./*Plasmodium* spp. and *Leucocytozoon* spp. in feral pigeons from northwest Italy, as well as the association between infection and host age or sex.

**Methods:**

Feral pigeons were collected during a regional culling programme from the Piedmont region (northwest Italy) and subjected to necropsy. Infections were detected from DNA extracted from the spleen following a nested PCR protocol. The association between sex or age and infection status was evaluated using the chi-squared test for independence or Fisher’s exact test.

**Results:**

Out of 51 animals, 15 were positive for *Haemoproteus/Plasmodium* spp. and eight for *Leucocytozoon* spp., with a significant difference between haemoparasites prevalence. There was no significant association between age or sex and infection status. The coinfection with different haemoparasites was very significant (p < 0.01), showing a greater relative risk to be infected by a second haemoparasite in birds already infected, in particular in male and in adult pigeons. DNA sequencing of *Leucocytozoon* spp. showed six different lineages in pigeons, and one of *Haemoproteus* and *Plasmodium,* respectively*.*

**Conclusions:**

Blood parasites are continuously circulating around the world, and the results presented in the paper suggest that cross infection of feral pigeons with haemoparasites typical of other migratory or nonmigratory bird species is possible. Moreover, the geographical location of Italy along the main migratory routes is a crucial factor to be considered for migratory birds, because they can be affected by blood parasites detected in feral pigeons, and vice versa.

## Background

Blood parasites have been subject of extensive research since the beginning of the 20th century [1]. The presence of haemoparasites in birds is very common, and it is estimated that 68% of all bird species are susceptible to haemosporidians [2,3]. Feral pigeons (*Columba livia*) are widely distributed in the world. Their number is increasing, especially in urban areas [4] . Haemoparasites in this species have been studied in several countries [5,6], but to the authors’ best knowledge no data on their presence in Italy are available. Therefore, the prevalence of *Haemoproteus* spp./*Plasmodium* spp. or *Leucocytozoon* spp. from pigeons culled by game wardens in the northwest of Italy has been determined. The possible associations between infection rate and different age or gender were also investigated. Moreover, considering that Italy is geographically situated on the main bird migratory routes, infection of feral pigeons with haemoparasites typical of other bird species could not be excluded, and vice versa.

## Methods

During the years 2010–2011, following the adoption of a regional animal containment programme (according to the D.G.R no. 74–6702 [08/03/2007] and subsequent amendments), 51 feral pigeons (28 males and 23 females), from the Piedmont region (northwest Italy) were captured with Larsen cage traps. The animals were euthanized with CO_2_, according to Italian National Bioethics Committee guidelines, and to law no. 157 (02/11/1992) and subsequent amendments, and subjected to a standard necropsy procedure. Spleen was collected and frozen at −20°C for biomolecular investigations. Age was determined by the colour of eyes and of the cere, according to the guidelines proposed by Beaman *et al.* [7].

Genomic DNA was extracted from sampled organs using a commercial DNA isolation kit (MACHEREY-NAGEL, Düren, Germany), according to the manufacturer’s protocol. The extracted templates were used for DNA amplification by mean of a thermal cycler (Gene Amp PCR System 2400, Perkin Elmer, Waltham, MA 02451, USA), using a specific primer set, as described by Scaglione *et al.* [8], exploiting the modified Hellgren *et al.* [9] protocol. A positive control DNA for each tested parasite was obtained from individuals with known infection obtained from eagles owl (*Bubo bubo*) tested by blood smears and PCR [10]. Each amplicon obtained from the second PCR step was visualized by means of agarose gel electrophoresis.

All positive samples were sequenced by Macrogen Europe (Amsterdam, the Netherlands), with the primers used for the second step of the nested PCR. Sequences were manually edited using BioEdit 7.0.5.3 and cytochrome *b* gene haplotypes, defined by a sequence difference of at least one base in the amplified fragment, were identified using the Nucleotide BLAST application of GenBank [11]. Mixed infections were recognized by the presence of double peaks on the electropherograms [12]. Whenever possible, the identities of the parasites involved were assessed by comparing the double peak patterns with previously known sequences of parasite haplotypes infecting pigeons obtained from GenBank and MalAvi. New sequences were given a name according to MalAvi nomenclature [13] and deposited in GenBank.

Statistical analysis was performed using GraphPad Prism software (GraphPad Inc., San Diego, CA). The association between sex or age and infection status was evaluated using the chi-squared test for independence or Fisher’s exact test. A p value less than 0.05 was considered statistically significant.

## Results

Out of the 51 feral pigeons tested by nested PCR, 15 (29.4%) resulted positive for *Haemoproteus/Plasmodium* spp. and 8 (15.7%) for *Leucocytozoon* spp. A highly significant difference between the haemoparasites prevalence (p < 0.001) was detected. There were no significant differences on infection status according to either bird age or sex.

The coinfection with both haemoparasites was very significant (p < 0.01), and a greater relative risk (RR, 7.2) to be infected by a second haemoparasite was recorded in already infected birds.

Regarding sex and age distribution of multiple infestation in pigeons associations were significant only in males (p < 0.05; RR 11.0) and in adult birds (p < 0.05; RR 10.0).

DNA sequencing of the eight PCR positive samples for *Leucocytozoon* spp. allowed identifying six different lineages: five of them were the same identified in Hooded crows investigated by the authors in the same area (LC2; LC3, LC4; LC5; LC6: Scaglione *et al.*, unpubl. data, [GenBank: KJ128987; KJ128989; KJ128990; KJ128991; KJ128988]), the other was a new lineage in feral pigeons (L-AEMO02). In two animals, two different lineages of *Leucocytozoon* (LC3-LC6 and LC2-LC4) were present at the same time. DNA sequencing of the 15 PCR positive samples for *Haemoproteus/Plasmodium* spp. showed the presence of the already described haplotypes P-SGS1 and H-HAECOL1. One *Leucocytozoon* sample and five *Haemoproteus*/*Plasmodium* samples that scored positive on the PCR analyses could not be sequenced, despite repeated amplification.

## Discussion

Haemoparasites in birds have been object of many investigations [1,14]. Usually blood samples are taken from free-living birds during bird ringing, in order to evaluate morphology of haemoparasite in blood cells and grading parasitaemia with light microscopy, and to identify different lineages by PCR. Blood samples and blood smears were not collected in this project because animals were captured in accordance with current national regulations through Larsen cage traps and immediately euthanized. After a standard necropsy, the spleen of each animal was tested by PCR for haemoparasites. Spleen, being a well perfused and easily removable organ, with an important role in destruction of parasitized cells [15], can be used as a substitute of blood, useful to identify an erythrocytic or megalomeront stage. In fact, during these stages of development, the spleen should test positive by PCR. Furthermore, megalomeronts can be detected anywhere in the bird organism, but most of all in the spleen [1].

Several studies on birds haemoparasites [16-18] demonstrate that young animals are more prone to be infected by haemoparasites, even if the majority of authors indicated that adults have a greater prevalence of infection by various groups of haemosporidians [19]. Other authors showed that there were no differences in the prevalence of bird infections in different age groups [20], as reported in the present investigation.

Numerous endogenous and exogenous factors may have a cumulative influence on the infection status of both sexes of the pigeons by these parasites, such as the host's hormones and humoral compounds, age and nutritional conditions, behaviour and habits, as well as the season of the year and ecological and physical features of the regions [6]. Usually, females are more parasitized because the reduction of the locomotion activity during the nesting period is a factor increasing the probability of their infection with haemosporidians [1].

In the present study, a significant association between sex and infection status was not detected. However, as the animals were captured from October to March, probably nesting related differences could not have been observed due to a reduced laying of eggs in this period. Moreover, in this species both male and female can hatch eggs.

Statistical differences in PCR detection of *Haemoproteus/Plasmodium* spp. and *Leucocytozoon* spp. infection may be due to variation in vector diversity and population size, and avian community composition [1,21,22].

The finding of avian haematozoa in pigeons implies the presence of ornithophilic vectors in Piedmont region and the susceptibility of this species to infection. Many bird hosts could be simultaneously infected by several species or strains of parasites [23-25]. According to the literature, *Haemoproteus* spp. is the most frequently observed blood parasite in birds, followed by *Leucocytozoon* spp. and *Plasmodium* spp. [26-28]. In *Columbidae* [5,6,28,29], prevalence for each of them is strictly correlated to the geographical area. Sequences analysed in this study revealed respectively positivities of 56.3%, 43.8%, and 6.3%, for the three species. The coinfection with *Haemoproteus* spp., *Plasmodium* spp. and *Leucocytozoon* spp. in tested samples showed that the presence of a haemoparasite predisposes to other haemosporidian infections, in particular in males and in adult birds.

Coinfections with two or more different malaria parasites are common in wild birds [30] and it is possible to find several haemosporidia lineages both in a population and in the individuals [9]. Beadell *et al.* [31] found mixed infections in 29/428 (6.8%) individuals in their study on the prevalence of two avian blood parasite genera (*Plasmodium* and *Haemoproteus*) in the Australo-Papuan region. In a recent study in New Zealand, double infections with *Plasmodium relictum* and *Plasmodium rouxi* were found in blackbirds [32]. There are discrepancies in the literature about the effect of coinfections with different strains of *Plasmodium*, although these are generally considered more virulent than infections with just a single strain [33-35]. Heavy parasitaemia (over 35% and up to 90% during peaks) was found in three species of experimentally infected passerines, but the authors did not note any significant effect in body mass [35]. In contrast, Marzal *et al.* [34] found a negative additive cost in body condition in individuals from a natural population of House Martins (*Delichon urbicum*) experimentally infected by two different *Plasmodium* strains [36].

The L-AEMO2 *Leucocytozoon* spp. lineage [GenBank: KJ152637] has been observed in *Aegypius monachus* in Spain (Pérez-Rodríguez *et al*., unpubl. data, [GenBank: HF543622]). The other lineages of *Leucocytozoon* spp. identified in feral pigeons have never been observed before in non-passerine birds. The L-CORNIX02 and L-CORNIX06 lineages have been previously observed in *Corvus corone* and *Corvus macrorhynchos* in Japan (Yoshimura *et al.* unpubl. data [GenBank: AB741499, AB741505]) and in *Corvus corone cornix* in Italy in the same area. The LC3, LC4 and LC5 lineages have been previously identified only in *Corvus corone cornix* in Piedmont. Similar sequences with a homology of 99% (473/479 bp) have been registered in *Pycnonotus jocosus* in Switzerland (van Rooyen and Christe, unpubl. data [GenBank: JX867112]).

The H-HAECOL1 *Haemoproteus/Plasmodium* spp. lineage has been previously recorded in *Columbida* [25,37] being repeatedly detected in pigeons and many other species of wild and domestic birds worldwide [38-42]. Reported infection rates were highly variable (range 18.8%-76.5%) but all the animals positive for *Haemoproteus* presented the H-HAECOL1 lineage 13, in contrast to Al-Barwari and Saeed [6], hypothesized that female pigeons are more prone than males to infection by H-HAECOL1. On the other hand, Senlik *et al.* [42] were unable to detect a significant difference in the infection rate of this parasite in terms of host sex, as already demonstrated in the present study.

Concurrent with the infection by H-HAECOL1, Dranzoa *et al.* [40] provided a figure of 29.4% for infection of the rock pigeons by *Plasmodium* parasites. In this study only one male pigeon was infected with *P. relictum* P-SGS1 lineage, not associated to other tested haemoparasites. The identified *Plasmodium* parasite has been observed worldwilde, mainly in passerine birds [43-45].

## Conclusion

Blood parasites are related in many characters and considered by many authors to have either benign or mild effects [39,18]. They are continuously circulating around the world and any variation in their prevalence, intensity and health impact, whether sex-, seasonal- or spatial-related, might depend on the susceptibility of the host species involved, their ages, habitats, as well as congruence, transmission, density and feeding habits of their vectors.

In the present paper the authors report novel host species for *Leucocytozoon* of corvids found in pigeons, and a novel *Leucocytozoon* species. *Leucocytozoon* parasites are not routinely found in pigeons. Moreover, the results hereby presented suggest that cross infection of feral pigeons with haemoparasites typical of other migratory or nonmigratory bird species is possible and should be further investigated and monitored. In fact, the geographical location of Italy along the main migratory routes is a crucial factor to be considered for migratory birds, because they can be affected by blood parasites transmitted by feral pigeons, and vice versa.

## Abbreviations

LC2: *Leucocytozoon*-CORNIX02
LC3: *Leucocytozoon*-CORNIX03
LC4: *Leucocytozoon*CORNIX04
LC5: *Leucocytozoon*-CORNIX05
LC6: *Leucocytozoon*-CORNIX06
L-AEMO02: *Leucocytozoon* AEMO02
H-HAECOL1: *Haemoproteus columbae* 1
P-SGS1: *Plasmodium relictum lineage* SGS1
